# A global analysis of implants and replacements of pacemakers and cardioverter-defibrillators before, during, and after the COVID-19 pandemic in Italy

**DOI:** 10.1007/s11739-023-03450-1

**Published:** 2023-11-07

**Authors:** Massimo Zecchin, Enrico Ciminello, Veronica Mari, Alessandro Proclemer, Antonio D’Onofrio, Gabriele Zanotto, Roberto De Ponti, Teresa Maria Capovilla, Paola Laricchiuta, Alessia Biondi, Letizia Sampaolo, Simona Pascucci, Gianfranco Sinagra, Giuseppe Boriani, Eugenio Carrani, Marina Torre

**Affiliations:** 1 Cardiothoracovascular Department, Cattinara Hospital, ASUGI and University of Trieste, Trieste, Italy; 2 Italian National Institute of Health, Rome, Italy; 3 Hospital Santa Maria Della Misericordia, Udine, Italy; 4 Monaldi Hospital, Naples, Italy; 5 Magalini Hospital, Villafranca Di Verona, Italy; 6 University of Insubria, Varese, Italy; 7 Cardiology Division, Department of Biomedical, Metabolic and Neural Sciences, University of Modena and Reggio Emilia, Modena, Italy

**Keywords:** Hospital discharge records, COVID-19 pandemic, Lockdown, Implantable cardioverter-defibrillator, Pacemaker, Replacement

## Abstract

At the beginning of the COVID-19 emergency, non-urgent surgical procedures had to be deferred, but also emergencies were reduced. To assess the global trend of pacemaker (PM) and implantable cardiac-defibrillator (ICD) procedures performed in Italy before, during, and after the first COVID-19 emergency, all the Italian hospital discharge records related to PM/ICD procedures performed between 2012 and 2021, sent to the National Institute of Health, were reviewed. Compared to 2019, in 2020, there was a reduction of first PM implants (52,216 to 43,962, −16%; *p* < 0.01), but not replacements (16,591 to 17,331, + 4%; *p* = 0.16). In particular, in April 2020, there was a drop of first implants (− 53,4% vs the average value of April 2018 and April 2019; *p* < 0.01), while the reduction of replacements was less evident (−32.6%; p = NS). In 2021, PM procedures increased to values similar to the pre-pandemic period. A reduction of ICD procedures was observed in 2020 (22,355, −7% toward 2019), mainly in April 2020 (− 46% vs April 2018/April 2019; *p* = 0.03). In 2021, the rate of ICD procedures increased (+ 14% toward 2020). A non-significant reduction of “urgent” procedures (complete atrioventricular block for PM and ventricular fibrillation for ICD), even in April 2020, was observed. In 2020, there was a reduction of first PM implants and ICDs, offset by increased activity in 2021. No decrease in PM replacements was observed, and the drop in “urgent” PM and ICD procedures was not statistically significant.

**Supplementary Information:**

The online version contains supplementary material available at 10.1007/s11739-023-03450-1.

## Introduction

Since March 11^th^ 2020, when the infection by SARS-CoV-2 was recognized by the World Health Organization as a pandemic, urgent decisions were necessary to reduce its transmission with important implications on daily life, including the organization of care. Italy was the first European country affected by the impact of COVID-19; to reduce the spread of the epidemic and to target resources for the management of patients affected by the SARS-CoV-2, non-urgent surgical procedures, including pacemaker (PM) implantations for sinus node dysfunction, II degree atrioventricular (AV) block (not advanced) without syncope, implantable cardioverter-defibrillator (ICD) implantations for primary prevention in stable low-risk outpatients, and the upgrade to cardiac resynchronization therapy (CRT) in stable patients, were considered deferrable [1]. Furthermore, in the same period, there was a significant reduction in admissions for cardiological emergencies [2]. The fear of infection combined with the lockdown-related restrictive measures and indications of Scientific Associations, following the governmental provisions to delay non-urgent procedures [3], resulted in a general decrease of surgical interventions, including cardiovascular implantable electronic devices (CIED) implantations, both in Italy and in other countries or regions [4–7]. To assess the nationwide impact of the COVID-19 pandemic on invasive procedures, the Italian Association of Arrhythmology and Cardiac Pacing (AIAC) launched a survey to evaluate the dynamic changes in arrhythmia care during the first five waves of COVID-19 in Italy [8]. However, more accurate data comparing overall national activity before, during, and especially after the most critical pandemic period are still lacking. In Italy, data from both public and private hospitalizations are routinely recorded in the Hospital Discharge Database (HDD) at the national level. This one is a standardized data collection that includes demographic and clinical information, such as diagnoses (principal and up to five secondary diagnoses, or comorbidities) and performed procedures (principal and up to five secondary procedures), labeled by the ICD-9-CM.

Aim of this work was to investigate the impact of COVID-19 pandemic and subsequent restrictions in healthcare system on PM and ICD implant volumes, by the exploration of HDD that the Italian National Institute of Health (NIH, Istituto Superiore di Sanità—ISS) receives from the Ministry of Health (MoH) on a yearly basis.

## Methods

Clinical data were extracted at the population level from the hospital discharge records (HDRs) database from 2012 to 2021. The ICD9-CM codes taxonomy defined in a previous study [9] was taken as a reference leading to the identification of ICD or PM procedures (Supplementary Table 1) and indications (Supplementary Table 2); unfortunately, according to present ICD9-CM codes, an accurate distinction between first implants and replacements of ICD is not feasible [9] (Supplementary Table 1).

The incidence of PMs (first implants and replacements) and ICDs (total procedures) were expressed as the number of procedures/year while implant rates (IR) as number of procedures/million of inhabitants. Italian population data by year were obtained from data publicly available at the Italian National Institute of Statistics (ISTAT, www.istat.it/en/ISTAT), the main producer of official statistics for citizens and policymakers, that operate independently in agreement with the academic and scientific community.

The diagnoses were identified according to ICD9-CM ninth revision codes; they were classified and sorted by indication, etiology, cardiac diagnosis, and non-cardiac diagnosis. Only the most frequent or clinically relevant diagnoses were displayed. For ICD implants, patients with a discharge diagnosis of ventricular tachycardia (VT) or ventricular fibrillation (VF) were considered treated for secondary prevention of sudden death (then replacements and first implants for primary prevention were excluded from this group).

The impact of the COVID-19 pandemic was explored by looking at hospitalization counts between 2018 and 2021 to compare the state of the Italian healthcare system in the cardiac implantable devices domain immediately before, during, and after the period of delay in non-urgent hospitalizations that occurred around April 2020. Both trend and conjunctural approaches to the time series were adopted. Time series were explored on a monthly basis, the independence of data was tested by Box–Pierce test, and significance in trends was checked by Mann–Kendall test [10–12]. The significance of the impact of delayed procedures in decreasing the number of hospitalizations in April 2020 was verified by outlier detection with Grubbs test, after testing for normality via Shapiro test. Significance level for all tests was fixed at *p* < 0.05. The statistical analysis was performed by Software R, version 2022.12.0 + 353 “Elsbeth Geranium” Release.

## Results

### Pacemakers

**Fig. 1:**
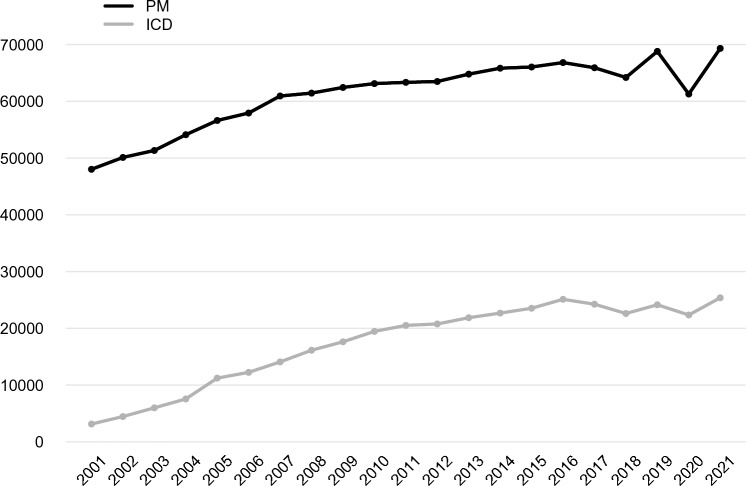
Total number of PM and implantable cardiac-defibrillator procedures per year from 2001 to 2021 in Italy. PM, pacemaker; ICD, implantable cardiac-defibrillator

From 2012 to 2019, the total annual number of PM procedures increased from 63,498 (1056/million inhabitants) to 68,807 (1150/million) (*p* = 0.03). In 2020, with 61,293 interventions (1027/million inhabitants), there was a statistically significant reduction of total procedures with respect to 2019 (–11%; *p* < 0.01 for outliers detection): first implants for PM decreased by 16% (from 52,216 to 43,962, *p* < 0.01), while PM replacements were almost stationary (+ 4%, from 16,591 to 17,331, *p* = 0.16). In 2021, there was an increase in volume similar to the pre-pandemic numbers (+ 13% for first implants; + 14% for replacements, compared to 2020) (Fig. 1).

**Focus on lockdown**

**Fig. 2:**
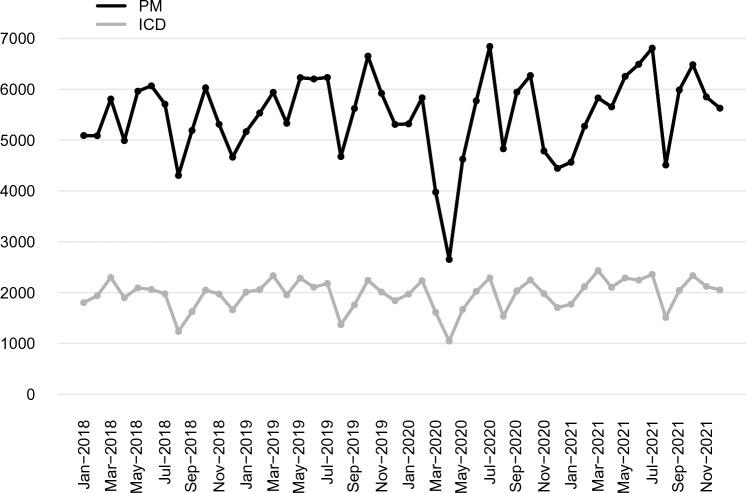
Total number of PM and ICD procedures per month from January 2018 to December 2021 in Italy. PM, pacemaker; ICD, implantable cardiac-defibrillator

**Fig. 3:**
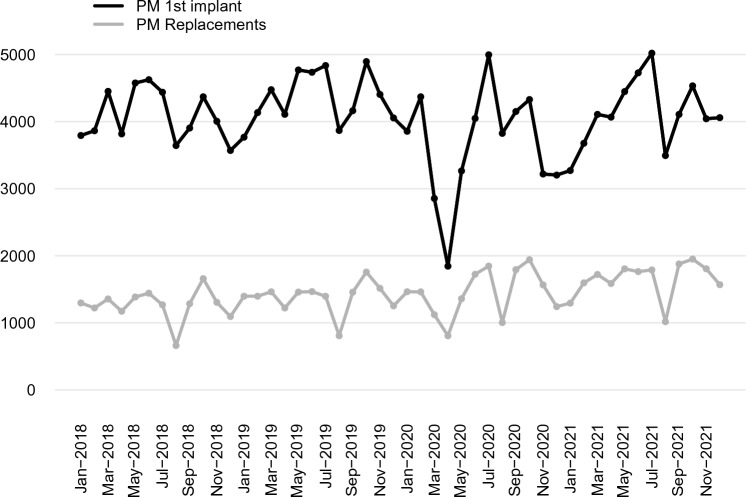
Number of first pacemaker implants and replacements per month from January 2018 to December 2021 in Italy. PM, pacemaker

A clear decrease in all PM procedures (Fig. 2) started in March 2020 and was remarkable (*p* < 0.01) in April 2020, compared to the average value of April 2018 and April 2019. This drop was mainly due to the reduction of first PM implants (− 53.4%; *p* < 0.01), while the reduction of replacements was less evident and not significant (− 32.6%; *p* = 0.17) (Fig. 3).

**PM indications**

In April 2020, compared to the average value of April 2018 and April 2019, a reduction of patients treated with first PM implant because of AV blocks (− 49%; *p* < 0.01), intraventricular blocks (− 44%; *p* = 0.04), syncope (− 57%; *p* < 0.01) and particularly sick sinus syndrome (− 64% *p* < 0.01) was observed (Table 1). Only the reduction of patients treated because of complete AV block (− 32%; *p* = 0.13) was not statistically significant.

**Table 1:** Pacemaker indications

Diagnosis	Apr-18	Apr-19	Jan-20	Feb-20	Mar-20	Apr-20	Jul-20	Apr-21	Outlier significance (Apr-2020)
Atrioventricular block, complete	941	1132	1068	1200	859	710	1287	1153	*p* = 0.13
Other heart block	143	158	132	146	98	53	185	155	*p* = 0.034
First degree atrioventricular block	55	49	51	56	43	12	72	59	*p* = 0.003
Mobitz (type) II atrioventricular block	355	426	405	451	280	165	436	458	*p* = 0.005
Other second-degree atrioventricular block	190	247	224	251	160	85	262	258	*p* = 0.002
Sick sinus syndrome	1012	1094	1010	1076	709	383	1389	937	*p* = 0.0027
Syncope	622	759	686	701	501	294	837	651	*p* = 0.004

#### ICD

For ICDs, a slow but statistically significant (*p* < 0.01) increase in procedure rates was observed from 2012 (20,774, 350/million) to 2019 (24,153, 403/million); afterward, there was a small reduction in 2020 (22,355, 375/million, − 7% towards 2019). In 2021, the rate of ICD procedures (25,384, 429/million) increased to values higher than those observed before the pandemic (+ 14% compared to 2020) (Fig. 1).

**Focus on lockdown**

**Fig. 4:**
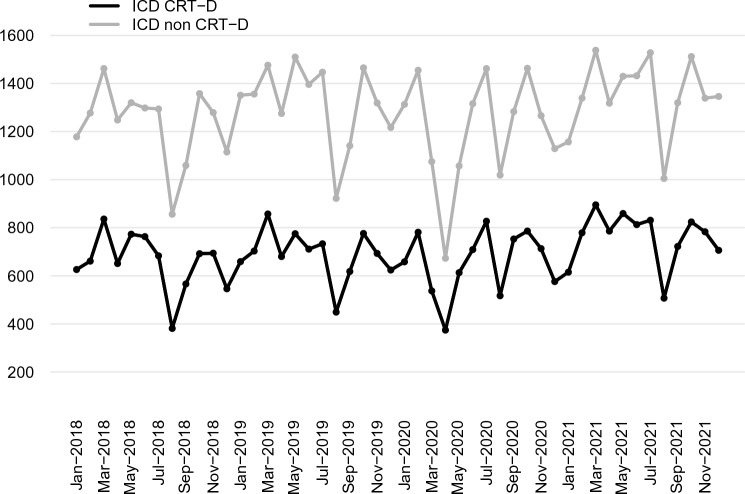
Total number of CRT-D and ICD non-CRT implants per month from January 2018 to December 2021 in Italy. CRT-D, cardiac resynchronization therapy defibrillator; ICD, implantable cardiac-defibrillator

In April 2020, compared to the average value of April 2018 and April 2019, there was an important drop in ICD procedures (− 46% p = 0.03) **(**Fig. 2**).** The reduction of CRT-D procedures was less striking (− 31%; *p* = 0.13) (Fig. 4).

**ICD indications**

A reduction of patients undergoing the procedures for all the indications to ICD was observed, so the relative proportion of cardiac and non-cardiac diagnoses was quite stable during the pandemic period. Also, the number of patients treated with ICD for secondary prevention (VT + VF) was reduced in April 2020 compared to April 2018 and April 2019 average values, but the difference was statistically significant for VT only (–52%; *p* = 0.02), while the reduction of patients treated because of VF did not achieve statistical significance (− 37%; *p* = 0.07) (Table 2).

**Table 2:** Implantable cardiac-defibrillators indications and diagnosis

Diagnosis	Apr-18	Apr-19	Jan-20	Feb-20	Mar-20	Apr-20	Jul-20	Apr-21	Outlier significance (Apr-2020)
Indication									
Ventricular fibrillation	81	98	93	82	76	43	92	62	*p* = 0.069
Ventricular tachycardia	247	280	288	306	251	167	306	265	*p* < 0.02
Etiology									
Congenital heart disease	4	3	4	4	1	1	10	4	*p* = 0.0428
Hypertensive heart disease	38	45	52	62	41	23	62	48	*p* = 0.0036
Hypertrophic cardiomyopathy	19	22	26	19	12	9	26	17	*p* = 0.15
Idiopathic cardiomyopathy	348	336	310	358	249	160	376	378	*p* = 0.0265
Ischemic heart disease	629	652	635	732	549	370	753	705	*p* = 0.0461

## Discussion

On March 8th 2020, all Northern Italy regions were quarantined and 3 days later, the lockdown was extended to the entire Italian territory.

According to governmental provisions, non-urgent procedures were postponed, while urgent procedures, as described in the recommendations of the National and international Scientific Associations, were allowed, providing the adoption of appropriate precautions and using the personal protective equipments.

Main results of our analysis, evaluating global trend of PM and implantable cardiac-defibrillator (ICD) procedures performed in Italy level before, during, and after the first COVID-19 emergency were:

1. In 2020, particularly in the month following the first lockdown, there was a reduction of PM and ICD implantations for both urgent and non-urgent procedures.
2. The reduction of most urgent procedures (complete AV block for PM and VF for ICD) was not statistically significant during the first lockdown.
3. In April 2020, first PM implants decreased compared to the same month of 2018 and 2019.
4. Compared to previous years, in 2020, PM replacements were stable, with only a slight reduction in April 2020.
5. In 2021, both first implants and replacements increased to values similar to the pre-COVID-19 emergency.
6. In 2020, a reduction of all ICD implantations was observed too, in particular during the first month of lockdown, despite a distinction between first ICD implants and replacements was not possible because of the lack of specific codes in ICD9-CM ninth revision system.
7. The reduction of CRT-D was less evident than in other ICD procedures. It is possible that patients with heart failure could have been considered less postponable than other patients treated with ICD for primary prevention of sudden death only.

Similar experiences were observed in other European countries. In Northwestern Greece, there was a decrease of 48% in de novo implantations and replacements of CIED in 2020, during the first lockdown period, compared to the corresponding period in 2019, but first PM implant and replacement rates did not change significantly during the year of COVID-19 pandemic, while ICD implantation rates declined by 31% [13].

In England, the reduction of PM implantations in the 3 months following the first wave of COVID-19 (started on March 20th, 2020) was 44% for PM and 45% for ICD [4].

In Catalonia, there was an absolute decrease of 56.5% in CIED implantations (54.7% in PM and 63.7% in ICD) after the declaration of the state of alarm in Spain on March 14th 2020 [6], with a rise following the peak of the pandemic (May to September 2020) to levels comparable to 2019; however, the total number of CIEDs implanted all through 2020 was significantly lower than 2019 (*p* < 0.0001).

Another comprehensive evaluation [14] of the Danish National Registry showed a less striking reduction, just 15% during the first lockdown. The reduction was similar for “acute” (for complete AV block) and not “acute” PM implantations and “acute” (cardiac arrest or ventricular arrhythmias) and “non-acute” ICD implantations.

Also in Germany, these trends were confirmed, with a drop in CRT-Ds similar to that observed by our study [15].

Few data on the period following the pandemic emergency are available. In the largest experience published so far, involving more than 177,000 patients from 1227 hospitals in China [16], the monthly CIED implantation decreased by 18% in 2020 compared with the pre-COVID-19 period but increased by 15.6% in 2021, like in the Italian experience (first PM implants + 13%, replacements + 14%, ICD + 14% compared to 2020).

In general, our study provides a detailed and precise analysis of what previously evaluated in Italy with a survey [8]. It also allows to compare the impact of COVID-19 in Italy with the experience from other European and non-European countries, and the drop (of roughly 50% considering the whole Italian territory) in both PM and ICD procedures during the first period of lockdown is a remarkable finding. However, the decline in activity was not completely offset by the end of the year, at least in most countries. In Italy, as expected, total PM replacements, which are not delayable for too long, did not change in 2020, while first PM implants and total ICD procedures were still reduced at the end of the year, compared to 2019. Such data can have several explanations, as the difficulty of “clear the backlog” by the hospitals, still managing COVID-19 emergency in most cases till the end of 2020 or the gradual, but not complete, recovery of confidence by patients in turning to health facilities.

In 2021, CIED implantations increased to values even higher than in the pre-COVID era, probably partly offsetting the reduction from the previous year.

The characteristics of patients at baseline did not change during and after the COVID-19 emergency for both PM and ICD patients, as the number of most PM and ICD indications reduced similarly, according to other experiences [17]. Considering the whole Italian territory, however, the drop of “urgent” PM and ICD procedures (for complete AV block and VF) was not statistically significant, so “necessary” procedures were reduced, but in a lesser amount than non-urgent implantations.

In some regional experiences, as in Veneto Region, the first Italian Region (together with Lombardia) affected by COVID-19, urgent PM implantations (defined as the presence of symptomatic sinus dysfunction or atrioventricular conduction disturbance that required admission to the emergency department and PM implantation within 1 week) significantly decreased during the 6 weeks after February 21st 2020 (from 122 to 88, − 28%, *p* = 0.02) and particularly after March 8th, in comparison to the 6 weeks before the first COVID-19 case [18].

Also in other Italian North-Eastern Centers, in March 2020, there were 51% fewer patients admitted for severe emergent cardiovascular diseases compared to March 2019, with a reduction of 50% of admissions for atrioventricular block/acute sinus node dysfunction [19]. Similar experiences were observed in other countries [6]**.** It is possible that the reduction of cardiac emergencies was more evident in regions with a greater impact of COVID-19.

There are limitations in making comparisons on the impact of COVID-19 pandemic among different countries or regions. As a matter of fact, even if the pandemic period was characterized by a striking increase in reports on literature related to the impact on admissions for cardiovascular diseases or emergencies [20, 21], the methods of reporting were not systematic, and different approaches (surveys, analysis of patients’ records, analysis of administrative data, etc.) were applied [7, 22–27].

### Study strengths and limitations

The strength of this study is the presentation for the first time of data considering the whole national implantation activity on a population level.

An important limitation is related to the accuracy of HDD recording and the use of ICD9-CM codes that cannot allow the distinction between first ICD implants and replacements. Finally, the aim of this study was limited to describe the impact of lockdown on clinical activity at national level. Further studies might focus on exploring differences at regional level.

## Conclusion

According to the Italian Hospital Discharge Records, during the COVID-19 pandemic period, there was a reduction in both non-urgent and urgent CIED procedures. However, the reduction of the most urgent procedures (complete AV block for PM and VF for ICD) was less striking and not statistically significant even in the first lockdown period (April 2020).

For PMs, there was a reduction of first implants in 2020, while PM replacements were unchanged; in April 2020, there was a drop in all PM procedures, but the reduction was not statistically significant for PM replacements in comparison with the average value of April 2018 and 2019. In 2021, both first implants and replacements increased to values even higher than before the COVID-19 emergency.

Similarly, a reduction in ICD implantations was observed in 2020, and in 2021, the rate of ICD procedures increased to values higher than those observed before the pandemic. In April 2020, compared to April 2018 and 2019, the reduction was statistically significant in general for all ICD procedures, but not for CRT-D.

### Supplementary Information

Below is the link to the electronic supplementary material.

Supplementary file1 (DOCX 289 KB)

## Data Availability

The data analysed in this study is subject to the following licenses/restrictions: the analysis of the data used in this study complies with the European General Data Protection Regulation (EU GDPR 2016/679) which authorised the processing of personal data relating to hospital discharge forms by ISS and other public institutions for reasons of public interest in public health. Written consent for participation was not required for this study, in accordance with national legislation and institutional requirements. The data that support the findings of this study could be available upon reasonable request submitted to MT [marina.torre@iss.it].

## Source of data

Italian Ministry of Health. National database of Hospital discharge records.
