# Supplementary material for: A global analysis of implants and replacements of pacemakers and cardioverter-defibrillators before, during, and after the COVID-19 pandemic in Italy
Source: Intern Emerg Med. 2023 Nov 7;19(1):107–14. doi: 10.1007/s11739-023-03450-1 (PMC10827813; doi:10.1007/s11739-023-03450-1)
Supplement: Supplementary file 1 — Supplementary file1 (DOCX 289 KB) [file 11739_2023_3450_MOESM1_ESM.docx]

Supplementary Table 1: ICD9-CM codes taxonomy for PM and ICD procedures

Supplementary Table 2: ICD9-CM codes taxonomy for PM and ICD indications
